# Broadband High-Gain Dual-Polarized Filtering Antenna Using a Partially Reflective Surface Lens for 5G Millimeter-Wave Sensor Applications

**DOI:** 10.3390/s25216742

**Published:** 2025-11-04

**Authors:** Yao Zhang, Huazhu Liu

**Affiliations:** 1 School of Electronic Engineering and Intelligence, Dongguan University of Technology, Dongguan 523000, China; zhangsantu@xmu.edu.cn; 2 Institute of Electromagnetics and Acoustics, Xiamen University, Xiamen 361005, China

**Keywords:** millimeter-wave sensor applications, filtering antenna, dual-polarized antenna, wideband antenna, partially reflective surface (PRS), lens antenna, high gain antenna

## Abstract

This paper presents a dual-polarized millimeter-wave filtering antenna based on a broadband partially reflective surface lens for gain improvement. It consists of a magneto-electric dipole (M-E dipole) as the source and a partially reflective surface (PRS) as the lens. The M-E dipole source antenna employs a dual-layer substrate structure, and its working principle is investigated by the circuit analysis method. A stub-loaded transmission line network is used to study the radiation characteristics of the source antenna, and the simulation results reveal that it has intrinsic integrated bandpass-type filtering response. The PRS lens is realized by designing a square high permittivity superstrate. By combining the source antenna and the lens, a wideband dual-polarized high gain cavity antenna is developed. The fabricated prototype has a measured impedance bandwidth of 33.3% (25–35 GHz), and a maximum in-band gain of 12.3 dBi. Above features make the proposed antenna a good candidate for 5G millimeter-wave sensor applications.

## 1. Introduction

Fabry–Pérot cavity (FPC) antennas have attracted increasing attention due to their moderate-to-high gain, simple feeding network, and planar structure. Such a cavity antenna usually consists of a ground plane, an antenna radiating source and a partially reflective surface (PRS) superstrate for antenna directivity enhancement. Many efforts have been devoted to improving the performance of the FPC antennas, such as utilizing quasi-curve ring reflectors to broaden the bandwidth [1], or employing multiple radiating sources to obtain multiple tilted beams, etc. [2].

On the other hand, filtering antenna integration design is a promising technique to be implemented in modern wireless communication systems. With the operating frequency increasing from microwave to millimeter-wave band, the insertion losses introduced by the passive filters or resonator circuits increase a lot correspondingly. Therefore, some traditional integration methods, including cascading the antenna radiator as the last stage of a filter network [3] or coupling parasitic filter components [4,5,6], are no longer efficient for millimeter-wave filtering antenna designs. Corresponding to this trend, some millimeter-wave filtering antennas have been proposed based on substrate-integrated waveguide (SIW) type [7,8], differential-fed patch type [9], reflectarray type [10] and tapered slot type [11]. In [12], a 60 GHz Fabry–Pérot cavity antenna driven by an SIW-fed filtering antenna was proposed for the first time with single-polarization operation.

In this paper, a wideband dual-polarized filtering FPC antenna for 5G millimeter-wave applications is proposed. The radiation characteristics of antenna source are revealed by investigating a one-port stub-loaded transmission line network. In order to provide multiple coherently wavefront radiations, a simple square high relative permittivity superstrate is deployed as the partially reflective surface. As a result, features such as wide bandwidth, high gain, dual-polarization, and integrated filtering performance are realized.

## 2. Source Antenna Working Principle

**Figure 1:**
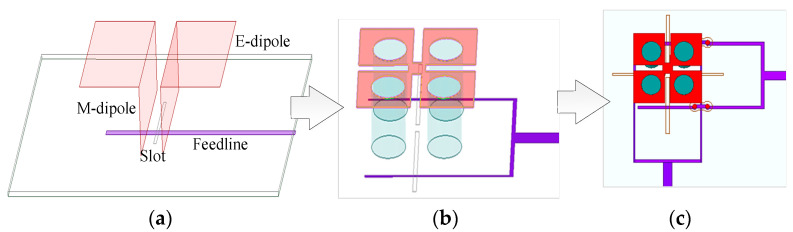
Design procedure of the proposed antenna: (**a**) Antenna I; (**b**) Antenna II; (**c**) Antenna III.

Figure 1 presents the design procedure of the source antenna. The proposed dual-polarized antenna is originated from an aperture-coupled M-E dipole antenna denoted as Antenna I. By ingeniously integrating an electric dipole and a magnetic dipole and leveraging their complementary resonances and orthogonal radiation characteristics, the ME-dipole antenna achieves excellent performance, including wide bandwidth, low cross-polarization level, stable radiation patterns, and consistent gain. As seen in Figure 1, Antenna I is composed of two horizontal metal patches (E-Dipole), a pair of vertically shorted patches (M-Dipole), a metal ground with a rectangular slot and a microstrip feedline. The metal ground with a rectangular slot is printed on the top side of a substrate while the microstirp feedline is printed on its bottom side. However, when the operating frequency increases to millimeter-wave band, the vertically shorted patches of this model cannot be realized using traditional fabrication process. Therefore, four symmetrical shorting vias are utilized to replace the pair of vertically shorted patches, forming the model denoted as Antenna II shown in Figure 1. Further, for dual-polarization operation, the feedline and the coupling slot of Antenna I are modified to a Y-shaped type and two separate short slots, respectively. Then Antenna III is developed in Figure 1.

**Figure 2:**
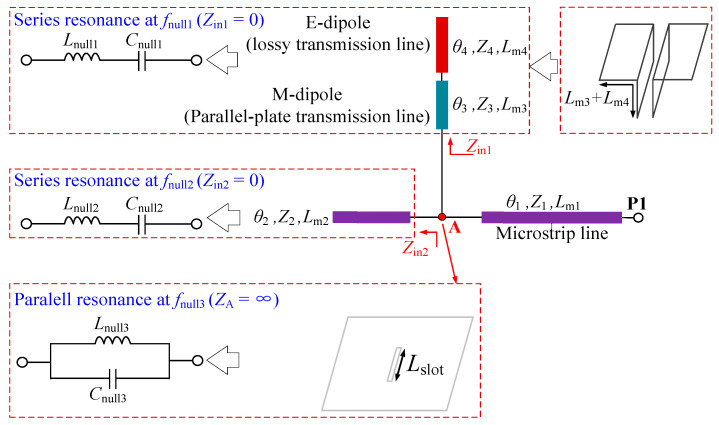
Equivalent circuits of the M-E dipole radiator, feedline, and coupling slot.

To reveal the working principle of the proposed Antenna III, circuit analysis method is used. As investigated in our previous work [13], the M-E dipole radiator can be regarded as a lossy transmission line (E-dipole) cascading a parallel-plate transmission line (M-dipole) [14]. Therefore, Antenna I can be approximated by a one-port stub-loaded transmission line network shown in Figure 2. In this network, the antenna microstrip feedline, M-dipole, and E-dipole are represented by a main microstrip line, a parallel-plate transmission line, and a lossy transmission line, respectively. The slot here is a dual-function component as either a microstrip-to-slotline transition structure [15] or a slot resonator [16]. The transmission characteristics of this one-port network as well as antenna radiation performance are studied and presented in detail as follows.

### 2.1. Two Intrinsic Radiation Nulls (Nulls 1, 2) Generated by the M-E Dipole Structure

**Figure 3:**
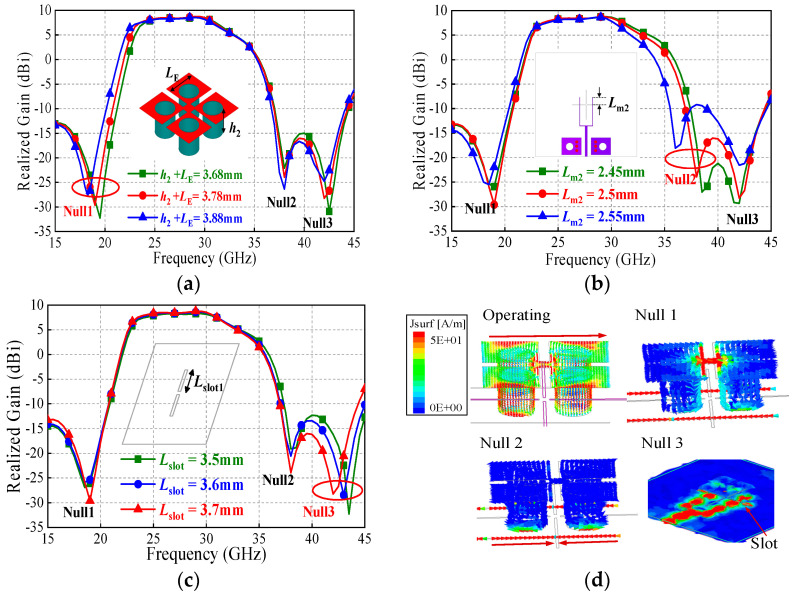
Frequency control: (**a**) Null 1; (**b**) Null 2; (**c**) Null 3 of the M-E dipole source without the PRS. (**d**) Current distribution.

In Figure 2, the values *θ*_i_, *Z*_i_, and *L*_mi_ denote the electrical length, input impedance, and physical length of corresponding parts (*i* = 1, 2, 3, and 4). Based on the transmission line theory [16], the input impedance of a transmission line terminated by a load Z_L_ can be obtained by

(1) $$Z_{\mathrm{in}}=Z_{0}\frac{Z_{L}+jZ_{0}\tan(\beta l)}{Z_{0}+jZ_{L}\tan(\beta l)}$$

where β is the wave number, which is related to the wavelength (λ) by the formula β = 2π/λ. Then *Z*_in1_ and *Z*_in2_, shown in Figure 3, can be expressed by

(2) $$Z_{\mathrm{in}1}=jZ_{3}\frac{Z_{3}\tan\theta_{3}-Z_{4}\cot\theta_{4}}{Z_{3}+Z_{4}\tan\theta_{3}\cot\theta_{4}}$$

(3) $$Z_{\mathrm{in}2}=-jZ_{2}\cot\theta_{2}$$

For the case *Z*_in1_ = 0 or Z_in2_ = 0, the point A is equal to be shorted and then the transmission zeros of the network could be generated. The Equations (2) and (3) can be simplified as

(4) $$\tan\theta_{3}\cdot \tan\theta_{4}=\frac{Z_{4}}{Z_{3}}$$

Parameters L_E_ and h_2_ are shown in Figure 1b.

(5) $$\cot\theta_{2}=0$$

If the impedance Z_3_ equals to Z_4_, then Equations (4) and (5) can be expressed by

(6) $$\theta_{3}+\theta_{4}=\frac{\pi }{2}+n\pi$$

(7) $$\theta_{2}=\frac{\pi }{2}+n\pi$$

It should be noted that the condition of Z_3_ equals to Z_4_ can be realized by optimizing the parameters R1 and d to change the impedance Z_3_ without affecting Z_4_. From (6) and (7), it is found that the electrical lengths L_m3_ + L_m4_ and L_m2_ are quarter wavelength at corresponding frequencies of the two transmission zeros. This indicates that at these two specific frequencies, the two stubs of the one-port network operate at series resonance, and thus the input impedance Z_in1_ = 0 or Z_in2_ = 0 is zero.

**Figure 4:**
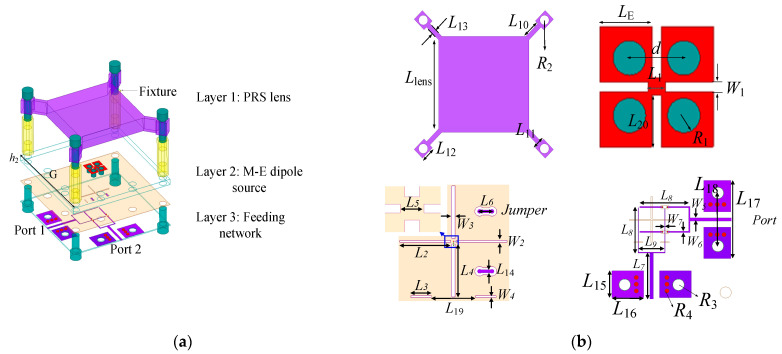
Configuration of the proposed antenna: (**a**) total view; (**b**) top view of PRS lens, top view of M-E dipole, top view of feeding part, bottom view of feeding part.

When this one-port circuit works as an antenna, two specific radiation nulls can be generated correspondingly. Simulations are conducted to verify the above analysis. Figure 4 illustrates the antenna-realized gains against corresponding parameters L_E_ + h_2_ (L_m3_ + L_m4_) and L_m2_. As observed in Figure 3a, there are three radiation nulls denoted as Null 1, 2, 3. When L_E_ + h_2_ increases from 3.68 mm to 3.88 mm, the frequency of the Null 1 decreases from about 19.5 GHz to 18 GHz, while the frequencies of radiation Nulls 2 and 3 remain almost unchanged. The current distribution on the antenna at the frequency of Null 1 is also presented. The red and blue regions in the figures denote strong and weak surface current areas, respectively. As seen, strong currents concentrate on the feedline, and partial currents are seen on the surface of M-dipole. The E-dipole part cannot be excited, and therefore the currents on it are weak.

Similarly, as shown in Figure 3b, the frequency of the Null 2 decreases from about 38.5 GHz to 36 GHz as Lm2 increases from 2.45 mm to 2.55 mm, while the frequencies of Nulls 1 and 3 are nearly unchanged. It is seen that the currents mainly concentrate on the feedline. The currents on the feedline divide into two parts and flow in the opposite direction. As a result, the current strength on the feedline under the slot is nearly zero and the input signal cannot be coupled to the radiator. It should be mentioned that, for the above two cases, the frequency of Null 3 is not affected, which is investigated below.

### 2.2. An Extra Radiation Null (Null 3) Generated by the Coupling Slot

In the proposed antenna, the slot operates as a dual-function component. At operating frequencies, it operates as a microstrip-to-slotline transition structure, which couples the input signal from the feedline to the M-E dipole radiator. However, under specific resonance condition, this slot can be excited as a slot resonator. Figure 2 shows its equivalent parallel LC resonance circuit. Herein, its fundamental resonant frequency can be calculated as

(8) $$f_{Null3}\approx \frac{c}{2L_{slot1}}$$

where c is the velocity of light in free space. This indicates that at this frequency *f*_Null3_, the slot does not function as a microstrip-to-slotline transition structure but as a non-radiative slot resonator. As a result, the signal cannot be transmitted from this slot to the M-E dipole radiator, and thus radiation Null 3 is generated. Figure 3c shows the antenna gains against the slot length parameter *L*_slot1_. As expected, the frequency of Null 3 can be individually controlled by tuning the value of *L*_slot1_ without affecting the frequencies of Nulls 1 and 2. As seen in Figure 3d, within the operating band, the antenna’s equivalent electrical length is half-wavelength at the operating frequency, resulting in a significant current distribution on the radiating structure. At the frequency of Null 1, the current distribution on the antenna radiator is weak, and the antenna does not function properly. At the frequency of Null 2, the currents on the feed line exhibit a reverse-phase cancellation effect at both ends of the slot. Furthermore, the operating current distribution on the antenna radiator is weak, causing the antenna to fail to operate effectively. At the frequency of Null 3, strong currents exist on the slot, while the current distribution on the antenna radiator remains weak. Thus, the antenna also fails to operate effectively.

## 3. Antenna Configuration

Figure 4 depicts the proposed antenna structure. It has three layers of substrates, which are used for a PRS lens, the M-E dipole antenna source, and the feeding network. Each layer of the structure is clearly shown in Figure 4b. Table 1 tabulates the antenna parameters. The square PRS lens is made of Rogers 4360 substrate with a thickness of 3.861 mm, loss-tangent δ of 0.0038, and relative permittivity of 6.4. Four pairs of mounting bolts are used as the fixture for the installation of the lens. The electric dipole (E-dipole) arms with length *L_E_* are printed on the top surface of the substrate Layer 2 while the magnetic dipole (M-dipole) arms are realized by inserting four shorting vias with radius R1. The four E-dipole arms are connected with each other and the M-dipole arms are also connected with the E-dipole ones. The substrate used for this part is Rogers 5880 with a relative permittivity of 2.2, a dielectric loss tangent of 0.0009, and a thickness of 1.575 mm. Four separate coupling slots (*L*_2_, *L*_4_) are seen on the top side, which couple the signal from the feedline to the ME-dipole radiator. Two jumper vias are inserted for the layout realization of dual-polarization operation. Due to the jumper vias, the reflection coefficients of the two antenna ports are inconsistent with each other. Therefore, we introduced two extra slots (*L*_3_) to maintain the balance. On the bottom side, it is seen that each feeding network consists of a 50 Ω microstrip line and a T-shape power divider. Two pairs of ground-connected pads are observed which are used for measurement. The substrate used for the feeding part is Rogers 3003 with a relative permittivity of 3.0, a dielectric loss tangent of 0.0013, and a thickness of 0.254 mm. Based on such a configuration, a wideband high dual-polarized FPC antenna is realized. The working mechanism of this antenna has been studied in detail in the following sections.

## 4. Partially Reflective Surface (PRS) Lens

**Figure 5:**
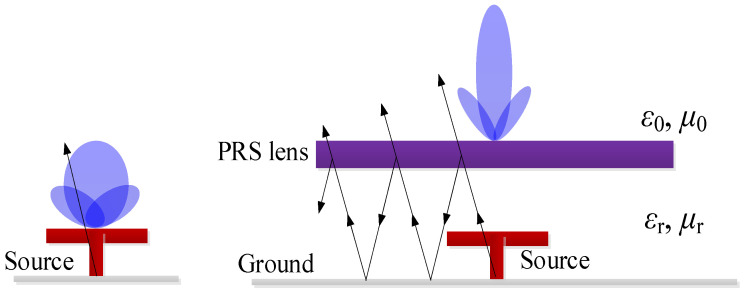
Realization of proposed source antenna with PRS lens.

As is known, due to the resonant response of the Fabry–Pérot cavity formed by the PRS and the ground plane when excited by an impinging plane wave, strong enhancement of directivity at a prescribed angle can be obtained [17]. As shown in Figure 5, when no lens is loaded on the source antenna, it achieves directional radiation with a wide-beam radiation pattern. However, when the lens is loaded, a portion of the electromagnetic waves radiated by the source antenna passes through the lens, while another portion is reflected by the lens back towards the ground plane. This reflected energy is then reflected again by the ground plane back towards the lens, initiating a multiple-reflection cycle. This process achieves an electromagnetic focusing effect. Consequently, the beamwidth of the radiation pattern narrows, and the gain increases. In this work, the PRS lens is realized by using a simple square high-permittivity superstrate. The size of the superstrate significantly affects the performance of the entire FPC antenna. For the proposed design, the bandwidth of the PRS should be no less than that of the M-E dipole source (33.3%). Based on the optimization results, the thickness and height of the superstrate are set to be 3.861 mm (0.38 λ_0_) and 10.5 mm (1.05 λ_0_).

**Figure 6:**
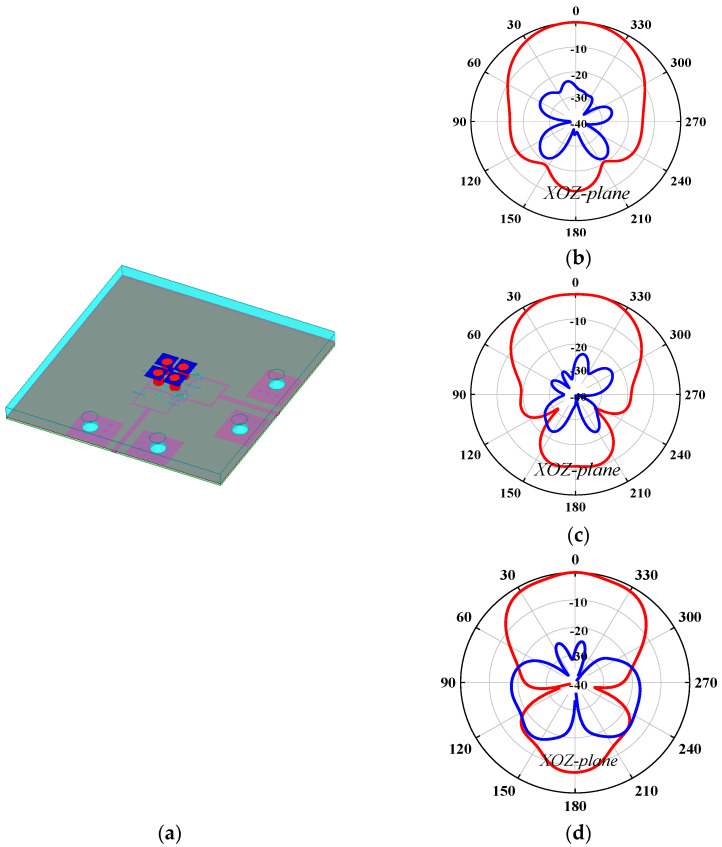
Antenna radiation patterns of the source antenna: (**a**) source antenna; (**b**) XOZ-plane radiation patterns at 25 GHz; (**c**) XOZ-plane radiation patterns at 30 GHz; (**d**) XOZ-plane radiation patterns at 35 GHz.

**Figure 7:**
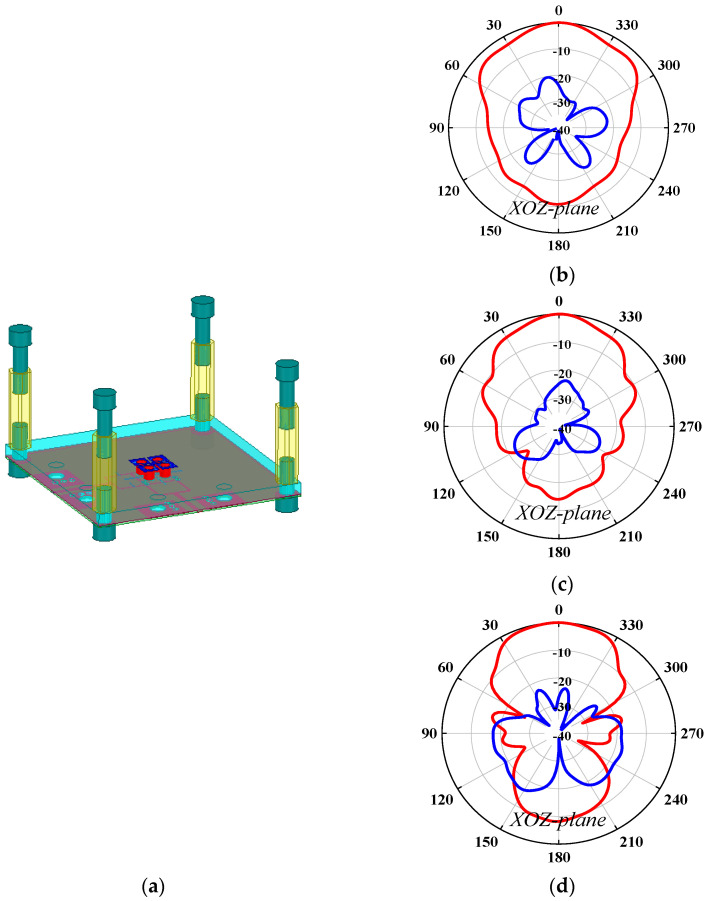
Antenna radiation patterns of the source antenna with four installation fixtures: (**a**) source antenna with four installation fixtures; (**b**) XOZ-plane radiation patterns at 25 GHz; (**c**) XOZ-plane radiation patterns at 30 GHz; (**d**) XOZ-plane radiation patterns at 35 GHz.

**Figure 8:**
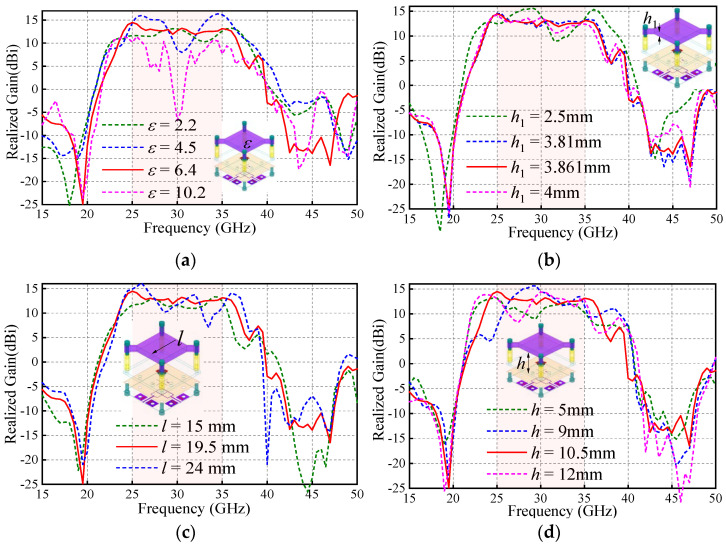
Antenna-realized gain against PRS lens: (**a**) dielectric constant *ε*; (**b**) length *l*; (**c**) thickness *h*_1_; and (**d**) height *h*.

The first problem is how to install the PRS lens. In this work, four nylon columns are used as the installation fixtures. Figure 6 and Figure 7 compare the antenna radiation patterns (25 GHz, 30 GHz, 35 GHz) of the proposed source antenna without and with the fixtures. As observed, the installation fixtures have large effects on the antenna radiation patterns. To investigate the PRS lens, Figure 8 shows the lens antenna-realized gain against the lens parameters including the dielectric constant *ε*, length *l*, thickness *h*_1_, and height *h*. To obtain a stable realized gain within the band 25 GHz–35 GHz (33.3%), the PRS lens values are finally selected as *ε* = 6.4, *l* = 19.5 mm, *h*_1_ = 3.861 mm, and *h* = 10.5 mm.

**Figure 9:**
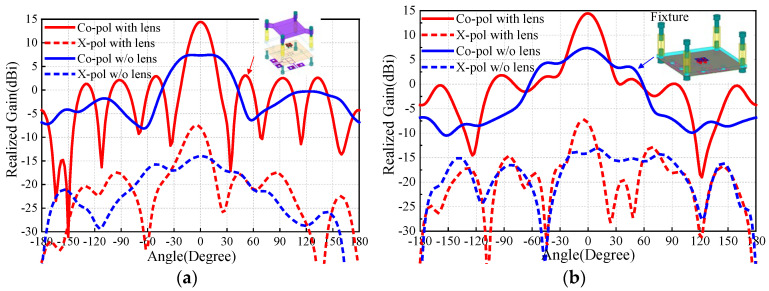
Simulation radiation patterns at 25 GHz with/without PRS lens: (**a**) E-plane; (**b**) H-plane radiation patterns.

**Figure 10:**
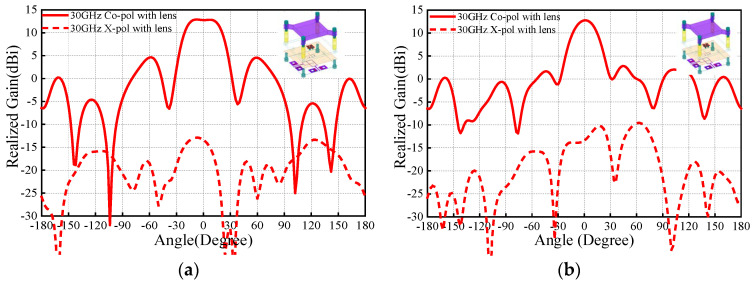
Simulation radiation patterns at 30 GHz with PRS lens: (**a**) E-plane; (**b**) H-plane radiation patterns.

**Figure 11:**
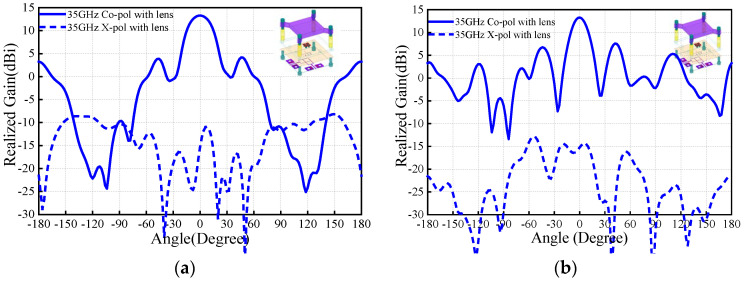
Simulation radiation patterns at 35 GHz with PRS lens: (**a**) E-plane; (**b**) H-plane radiation patterns.

Figure 9 compares the E-/H-plane radiation patterns of the antenna source with and without the PRS lens at 25 GHz. As seen, for the antenna without PRS lens, broadside radiation is realized with maximum simulated realized gain of 7.4 dBi. It should be mentioned that the blue solid/dotted lines shown in Figure 9 are the results of the M-E dipole antenna source with the fixture, which is used for installation of the PRS lens. This fixture has effect on the antenna radiation pattern, and therefore the side-lobe level of the H-plane is relatively high. With regard to the case with PRS lens, maximum realized gain of 14.5 dBi is obtained, indicating more than 6.1 dB gain enhancement is realized. Correspondingly, the E-/H-plane 3 dB beamwidths reduces from 66° and 57.3° to 20° and 24°. The co-polarized fields are more than 20 dB stronger than its corresponding cross-polarized counterparts. These results demonstrate that satisfactory beam-focused effect is realized. To verify the wideband performance of the PRS, the E-/H-plane radiation patterns of the antenna with PRS lens at 30 GHz and 35 GHz are illustrated in Figure 10 and Figure 11. As observed, around 13.5 dBi broadside-realized gains and low cross-polarized fields are obtained.

## 5. Antenna Implementation

**Figure 12:**
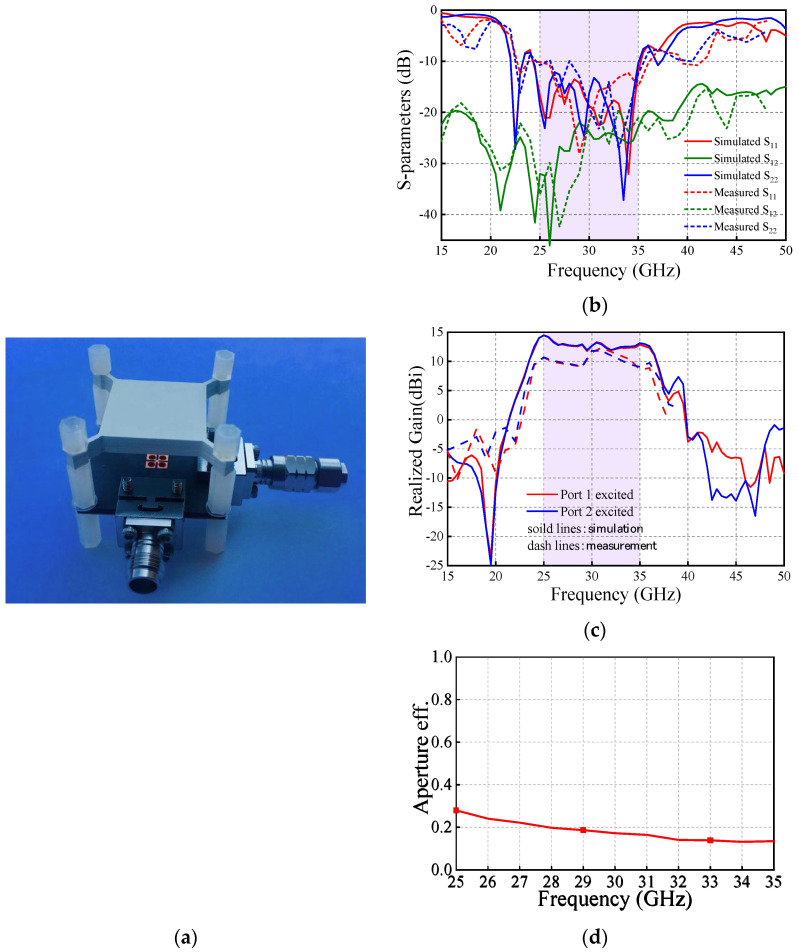
Antenna measurement: (**a**) Antenna fabrication prototype and simulation and measurement results including (**b**) S-parameters and (**c**) realized gains. (**d**) Aperture efficiency.

Based on the M-E dipole antenna source and the PRS lens, a Fabry–Pérot cavity antenna is developed. The optimization was performed using high frequency structural simulator (HFSS 2014), and the measurement was accomplished by Agilent N5227A network analyzer and NI system. Figure 12 illustrates the antenna prototype and the simulation and measurement results. As observed, the measured antenna impedance bandwidth (|S11| < −10 dB) is 33.3% (25–35 GHz). Within this band, better than 20 dB measured isolation between the two ports is obtained. The aperture efficiency is about 20%. The measured in-band gain ranges from 9.1 dBi to 12.3 dBi, whereas the simulated one is from 13.0 dBi to 14.5 dBi.

To address the advantages of the proposed work, the comparison results with counterparts are tabulated in Table 2. The filtering M-E dipole in [13] operates at microwave band, whereas the others [9,12,18] are millimeter-wave designs. In [12], a single-polarized filtering FPC antenna was firstly proposed with high gain and high frequency selectivity. Compared to the above designs, this work simultaneously realized dual-polarization, wide bandwidth, high gain, and integrated filtering performance.

## 6. Conclusions

In this paper, a wideband dual-polarized millimeter-wave filtering Fabry–Pérot cavity (FPC) antenna has been developed based on an M-E dipole source and a PRS lens. Without any filter circuit, the proposed antenna-realized inherent bandpass-type filtering radiation response. The fabrication prototype has a measured bandwidth of 33.3% (25–35 GHz). The measured in-band gains range from 9.1 dBi to 12.3 dBi.

## Figures and Tables

**Table 1:** Antenna parameters (mm).

*L*_1_	*L*_2_	*L*_3_	*L*_4_	*L*_5_	*L*_6_	*L*_7_	*L*_8_	*L*_9_	*L*_10_
0.7	3.7	1.5	3.9	0.4	0.9	8.5	8.1	4.6	3.7
*L*_11_	*L*_12_	*L*_13_	*L*_14_	*L*_15_	*L*_16_	*L*_17_	*L*_18_	*L*_19_	*L*_20_
2.5	3	1	0.22	4.8	5.6	14	9.5	3.2	2.05
*L_lens_*	*h*_2_	*W*_1_	*W*_2_	*W*_3_	*W*_4_	*W*_5_	*W*_6_	*W*_7_	*d*
19.5	1.575	0.4	0.15	0.25	0.15	0.6	0.22	0.1	2.3
*L_E_*	*d*	*G*	*R*_1_	*R*_2_	*R*_3_	*R*_4_			
2.2	2.3	25	0.7	1	1	0.4			

**Table 2:** Comparison of four counterparts.

	Antenna Type/Layer	Polar	FBW (%)	Radiation Null	Gain (Sim)
[13]	M-E dipole/-	Dual	69.3	3	~8 dBi
[9]	Patch/5	Dual	20	3	~7 dBi
[18]	FPC/2	Single	22.2	0	~13 dBi
[12]	FPC/4	Single	15.7	4	~15 dBi
This work	FPC/3	Dual	33.3	3	~13 dBi

## Data Availability

Data are contained within the article.

## Abbreviations

The following abbreviations are used in this manuscript:

M-E dipole	Magneto-electric dipole
PRS	Partially reflective surface
FPC	Fabry–Pérot cavity

## References

[1] Guo Q.Y., Wong H. (2020). A millimeter-wave Fabry–Pérot cavity antenna using fresnel zone plate integrated PRS. IEEE Trans. Antennas Propag..

[2] Guo Q.Y., Wong H. (2019). Wideband and high-gain Fabry–Pérot cavity antenna with switched beams for millimeter-wave applications. IEEE Trans. Antennas Propag..

[3] Mao C.X., Gao S., Wang Y., Chu Q.X. (2015). Multi-mode resonator-fed dual polarized antenna array with enhanced bandwidth and selectivity. IEEE Trans. Antennas Propag..

[4] Yang W.C., Chen S., Xue Q., Che W.Q., Shen G.X., Feng W.J. (2019). Novel filtering method based on metasurface antenna and its application for wideband high-gain filtering antenna with low profile. IEEE Trans. Antennas Propag..

[5] Lin X., Weng Z., Hong Y., Zhang Y. (2024). A Wideband Circularly Polarized Dipole Antenna with Compact Size and Low-Pass Filtering Response. Sensors.

[6] Hsieh C.Y., Wu C.H., Ma T.G. (2015). A compact dual-band filtering patch antenna using step impedance resonators. IEEE Antennas Wireless Propag. Lett..

[7] Chu H., Chen J.X., Guo Y.X. (2015). A 3-D millimeter-wave filtering antenna with high selectivity and low cross-polarization. IEEE Trans. Antennas Propag..

[8] Hu H.T., Chan C.H. (2021). Substrate-integrated-waveguide-fed wideband filtering antenna for millimeter-wave applications. IEEE Trans. Antennas Propag..

[9] Yang S.J., Pan Y.M., Shi L., Zhang X.Y. (2020). Millimeter-wave dual-polarized filtering antenna for 5G application. IEEE Trans. Antennas Propag..

[10] Wu G.B., Zeng Y.S., Chan K.F., Chen B.J., Qu S.W., Chan C.H. (2020). High-gain filtering reflectarray antenna for millimeter-wave applications. IEEE Trans. Antennas Propag..

[11] Hu M.Y., Yu Z.Q., Xu J., Lan J., Zhou J.Y., Hong W. (2021). Diverse SRRs loaded millimeter wave SIW antipodal linearly tapered slot filtenna with improved stopband. IEEE Trans. Antennas Propag..

[12] Hu H.T., Chan K.F., Chan C.H. (2022). 60-GHz Fabry-Perot cavity filtering antenna driven by an SIW-fed filtering source. IEEE Trans. Antennas Propag..

[13] Zhang Y., Zhang X.Y., Liu Q.H. (2021). Dual-polarized filtering magneto-electric dipole antenna utilizing intrinsic highpass filter network and integrated lowpass filter network. IEEE Trans. Antennas Propag..

[14] Pramudita A.A. Input impedance model of planar dipole antenna for Wireless Body Area Network (WBAN). Proceedings of the Asia-Pacific Conference on Communications (APCC).

[15] Knorr J.B. (1974). Slot-line transitions. IEEE Trans. Microw. Theory Techn..

[16] Pozar D.M. (1998). Microwave Engineering.

[17] Hosseini A., Capolino F., De Flaviis F., Burghignoli P., Lovat G., Jackson D.R. (2014). Improved bandwidth formulas for Fabry-Pérot cavity antennas formed by using a thin partially-reflective surface. IEEE Trans. Antennas Propag..

[18] Goudarzi A., Honari M.M., Mirzavand R. (2021). A millimeter-wave Fabry-Perot cavity antenna with unidirectional beam scanning capability for 5G applications. IEEE Trans. Antennas Propag..

